# Supplementary material for: Empirical methods for controlling false positives and estimating confidence in ChIP-Seq peaks
Source: BMC Bioinformatics. 2008 Dec 5;9:523. doi: 10.1186/1471-2105-9-523 (PMC2628906; doi:10.1186/1471-2105-9-523)
Supplement: Additional File 1 — A variety of html documents from the USeq web site detailing the available applications, their best usage, output file type descriptions, command line menus, etc. [file 1471-2105-9-523-S1.zip › USeqUserGuides/JavaDoc.zip!/JavaDoc/trans/roc/package-frame.html]

trans.roc


trans.roc

Classes 
  
Bin
  
ControlGene
  
ExonScoreExtractor
  
ExtractNonOverlappingWindows
  
ExtractSaveSgrScores
  
ExtractScoresForRoc
  
Gr
  
GrComparator
  
MethodTester
  
ParsePatternedWindows
  
ParseSgrsForParticularRegions
  
ParseSgrsForParticularScores
  
Positive
  
PositiveComparator
  
PWMScoreWindows
  
RocWindow
  
RocWindowScanner
  
ScoreParsedBars
  
Sgr
  
SgrBacCounter
